# Supplementary material for: Tumor Protein D53 (TPD53): Involvement in Malignant Transformation of Low-Malignant Oral Squamous Cell Carcinoma Cells
Source: Biomedicines. 2024 Nov 28;12(12):2725. doi: 10.3390/biomedicines12122725 (PMC11727615; doi:10.3390/biomedicines12122725)
Supplement: Supplementary file 1 [file biomedicines-12-02725-s001.zip › biomedicines-3314158-supplementary.pdf]

Supplementary Figures

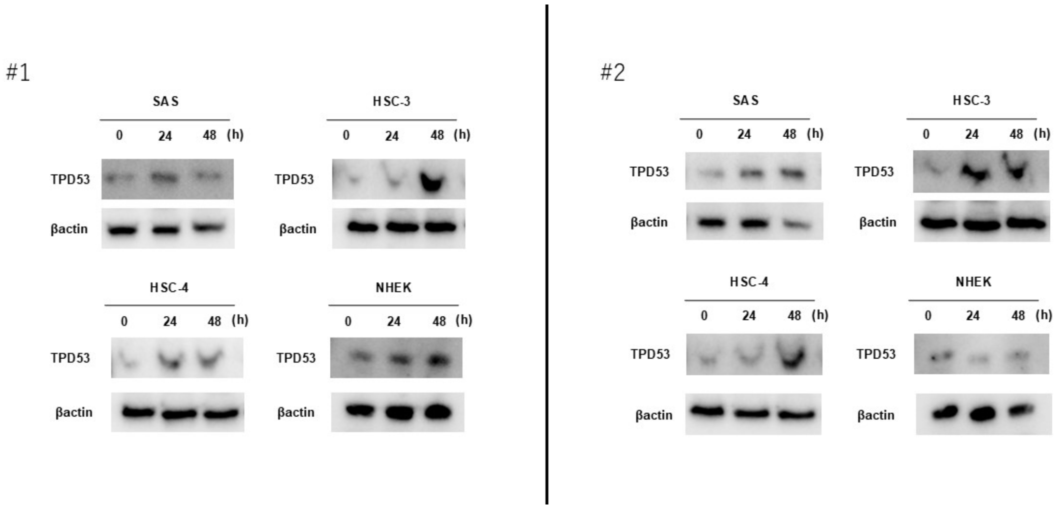

Supplementary Figure S1. Expression of tumor protein D53 (TPD53) in oral squamous cell carcinoma (OSCC) and normal human epidermal keratinocyte (NHEK) cells. The other 2 results of western blotting in thrice independent experiments are shown.

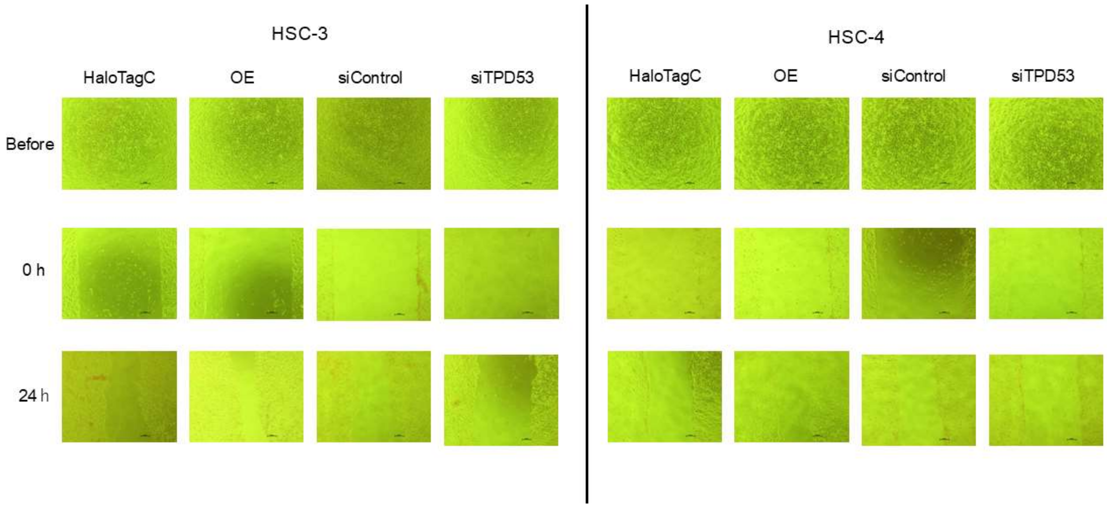

Supplementary Figure S2. Unstained Wound healing assay. HaloTag control vector (HaloTagC), HaloTag-TPD53 expression vector (OE), control siRNA (siControl) and siRNA TPD53 (siTPD53) were transfected into HSC-3 and -4 cells, and were grown for 24 h. The cells were re-seeded at a 24-well tissue culture plate. The microscopic images by a phase-contrast microscope were taken at the time after 24 h from re-seeding (Before), immediately after scratching (0 h) and after another 24 h (24 h), without fixing or staining.

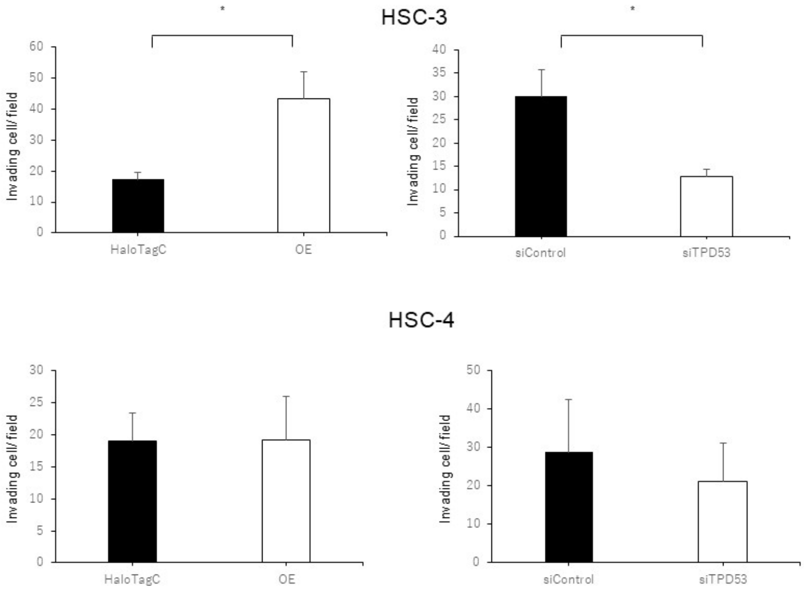

Supplementary Figure S3. Cell invasion assay. The assay was carried out using a commercially available kit (see Material and Methods). Thereafter, photographs were taken using a microscope and a microscopic CCD camera. The invading cells were counted by ImagJ.

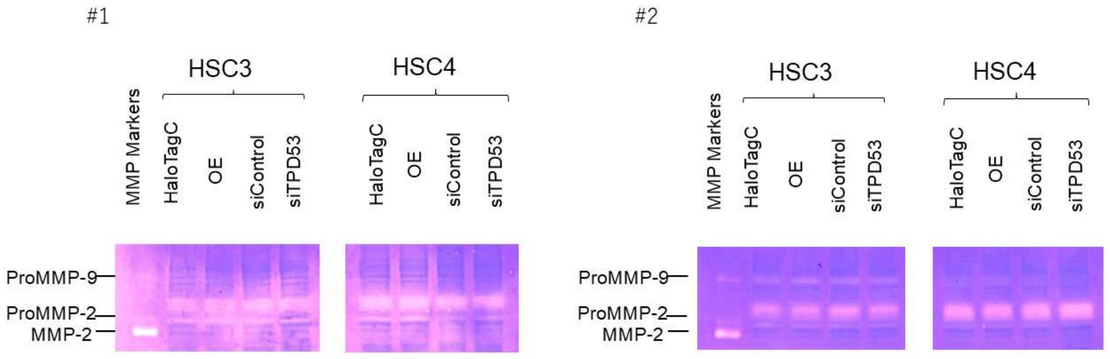

Supplementary Figure S4. Gelatin zymography for MMP activities. The other 2 results in thrice independent experiments are shown.

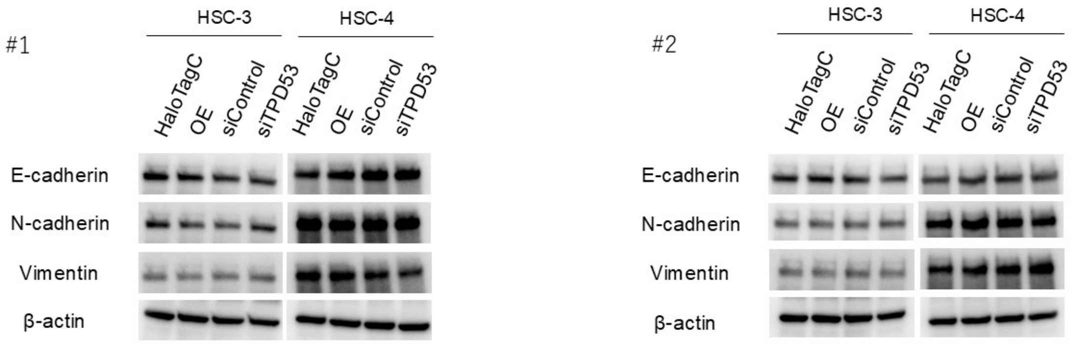

Supplementary Figure S5. Western blot analysis for assessing EMT. The other 2 results in thrice independent experiments are shown.

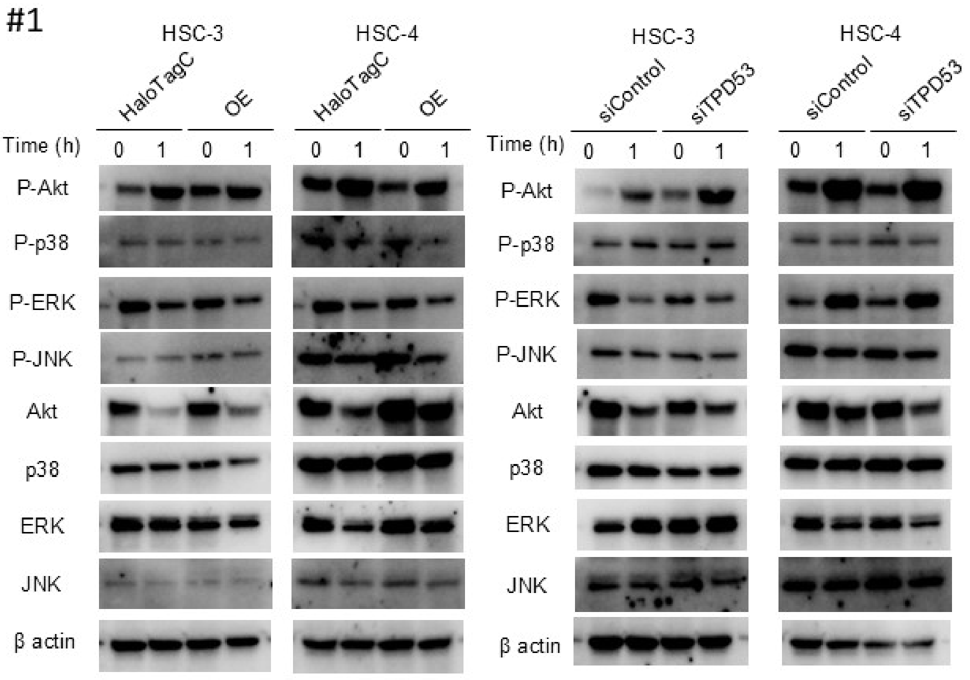

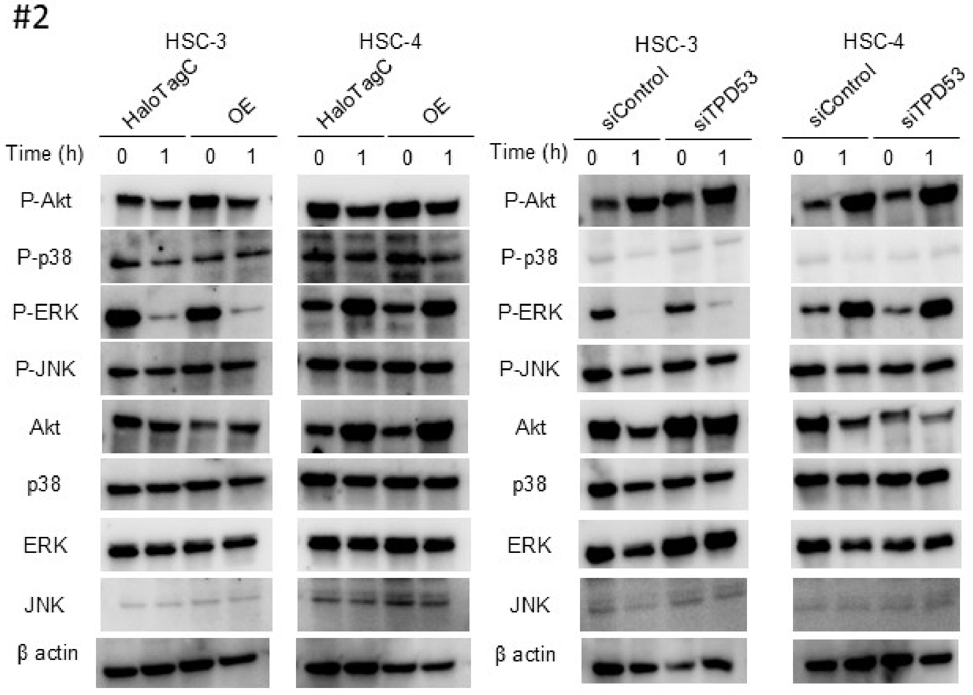

Supplementary Figure S6. Western blot analysis of total and phosphorylated Akt, p38, extracellular signal-regulated kinase (ERK), and c-Jun N-terminal kinase (JNK). The other 2 results in thrice independent experiments are shown.

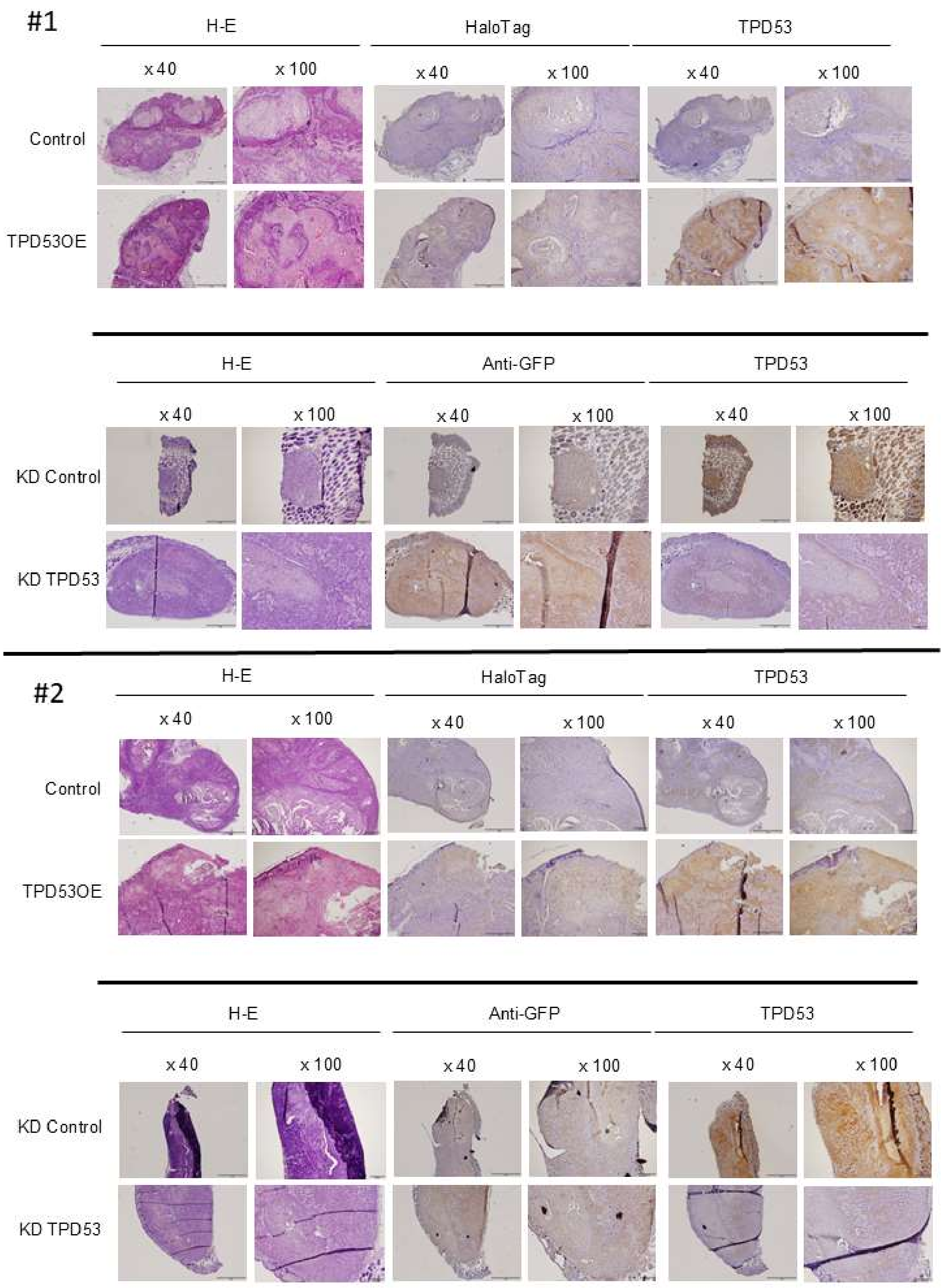

Supplementary Figure S7. Histopathological images of tumors. Images of the periphery and center of tumors, not shown Figure 7 in repeated thrice, are represented. Bars, 1 mm (40×) and 200 µm (100×).
